# Real-Time Clinical Decision Support System with Data Stream Mining

**DOI:** 10.1155/2012/580186

**Published:** 2012-07-18

**Authors:** Yang Zhang, Simon Fong, Jinan Fiaidhi, Sabah Mohammed

**Affiliations:** ^1^Department of Computer and Information Science, University of Macau, Macau; ^2^Department of Computer Science, Lakehead University, Thunder Bay, Canada

## Abstract

This research aims to describe a new design of data stream mining system that can analyze medical data stream and make real-time prediction. The motivation of the research is due to a growing concern of combining software technology and medical functions for the development of software application that can be used in medical field of chronic disease prognosis and diagnosis, children healthcare, diabetes diagnosis, and so forth. Most of the existing software technologies are case-based data mining systems. They only can analyze finite and structured data set and can only work well in their early years and can hardly meet today's medical requirement. In this paper, we describe a clinical-support-system based data stream mining technology; the design has taken into account all the shortcomings of the existing clinical support systems.

## 1. Introduction

Data Stream Mining is the process of extracting useful information from continuous, rapid data streams. Data Stream Mining is a very broad concept and it involves many technical areas such as classification, detection, and clustering. In this paper, the authors mainly focus on data stream classification, because clinical support systems usually require real-time medical prediction and classification based on multivariate data that have many attributes and terms. One of the main algorithms in clinical support system is decision tree and that will have to be improved in order to handle new applications where data feed in as streams instead of a structured data archive. Traditional decision tree is known which has a limitation that its learning requires scanning through the whole database many times. Data stream is a new data concept where segments of data can only be processed one pass at a time. The corresponding decision tree will have to offer a best possible decision over such unbounded data stream, whenever it is being queried. Since about year 2000, there have been progressively a handful of decision tree algorithms for data stream mining emerged, such as Very Fast Decision Tree (VFDT) [1] and Concept Adapting Very Fast Decision Tree (CVFDT) [2]. However, these decision trees may not be directly applied for medical use, many supporting tasks are needed, and they will be introduced in this paper.

In the commercial market there exist a number of clinical decision support systems. However, most of them are based on traditional technologies, such as traditional decision tree, Bayesian Network and Neural Network. These technologies are mature and reliable, but they all required consuming the data as static database. Each model refreshing may require going through the whole dataset again. More importantly, most of these traditional systems can only make diagnosis, they cannot make prediction. There are very few systems that adopt data stream mining algorithms. Moreover these systems are either doing detection or diagnosis. The most similar clinical decision system from our survey based on data streams is the one developed by IBM (http://www.ibm.com/us/en/). It is a pioneer that is capable of making prediction from medical data streams. The IBM clinical decision support system is compared with our proposed system in this paper.  Table 1 shows some typical existing clinical decision support systems that are available from the latest research work from the academics.

There are some defects in the above clinical decision support systems mainly on traditional data mining algorithms. The limitations are described in Table 2.

## 2. Proposed Solution

**Figure 1:**
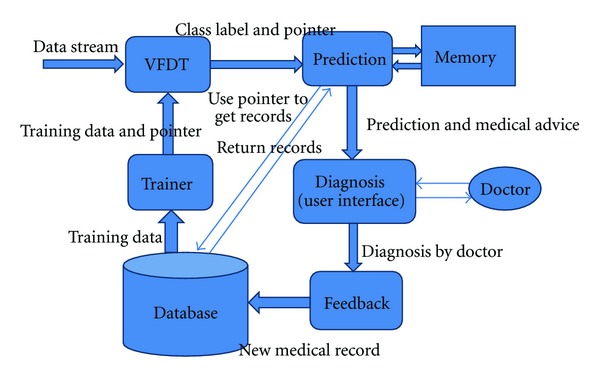
Logical structure of our design.

In order to innovate an effective real-time clinical decision support system, we should use an algorithm that can analyze data efficiently and accurately. Traditional decision tree may be a good choice for structured database; however, it cannot handle continuous rapid data streams. To solve this problem, we must use a more powerful and advanced decision tree algorithm such as Very Fast Decision Tree (VFDT). VFDT was developed by Domingos and Hulten. It is a decision tree algorithm based on Hoefding Tree. It uses fixed size memory and time to analyze every sample (data at a time point). VFDT's efficiency is much higher than traditional decision tree. It can handle large amounts of continuous data which we called data streams. However, if we use only the original VFDT, we can only know the current situation (illness), we cannot predict the situation in few minutes (hours). So in our design, pointers are added on the VFDT's leaf nodes. For each leaf nodes, it can be looked as a class label and it indicates a certain kind of medical situation (illness). Each leaf node has one or several pointers. These pointers are added by the learning algorithm in the training process. Each pointer has a unique value and each pointer represents a unique medical record which is similar with the current situation in database. And there is a mapping table in our system. This table stored every medical record, physical address (path) and its pointer value. So when VFDT directs a medical stream to a leaf node, we can use the pointer in this leaf node to search for the mapping table then it uses the physical index addresses to get these similar history medical records directly. Then we can use these similar (or relevant) records to make a prediction. The illustration of the system logical structure is shown in Figure 1.

### 2.1. Classification Process

**Figure 2:**
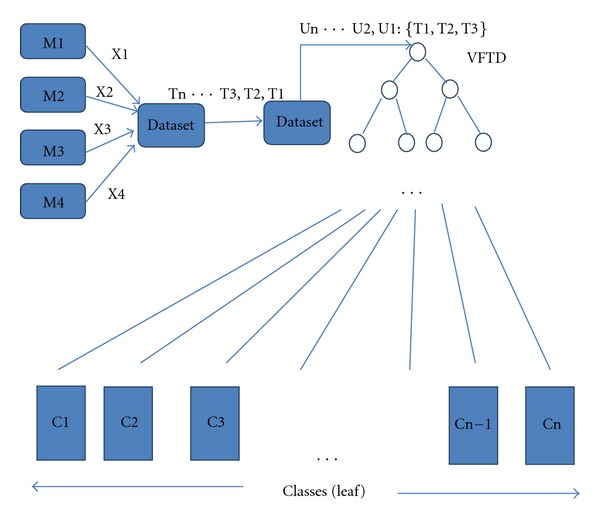
Classification by VFDT.

The description of how the stream classification algorithm (VFDT) works in the proposed model is explained via an example.  Figure 2 shows the outline of the classification example. For example, at time point T1 the medical data from four different kinds monitors (M1, M2, M3, M4) are given as blood pressure (M1) is X1, ECG (M2) is X2, EEG (M3) is X3, and body temperature (M4) is X4. So, data set at T1 is a vector of numeric measurements from the sensor devices, {X1, X2, X3, X4}. The data stream includes many time points in a data set like T1, T2, T3…Tn. We can put several consecutive time series points into a unit such as U1: {T1, T2, T3}, U2: {T4, T5, T6}, and U3: {T7, T8, T9}. So the data stream will be divided into multiple captures and every capture is a data unit. Every data unit represents the trend of illness corresponding to a period of time. The VFDT analyzes every data unit and indicates what the current situation is.

**Figure 3:**
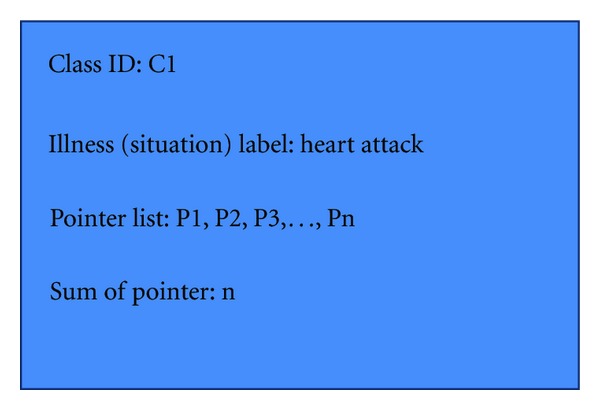
Leaf node structure.

C1, C2… Cn are leaf nodes of the VFDT tree, each of them indicates one class. Each class indicates one kind of illness or medical situation in general. When the data stream is analysed by the VFDT, the current situation will be directed to a leaf node (certain illness class), and the information in this leaf node will send it to prediction algorithm. The structure of leaf node (class) is shown in Figure 3.

P1, P2, P3,… Pn are location pointers, they represent digital medical records which are similar to the current situations that have happened in the past. Suppose the VFDT directs the current situation to the leaf node C1, then the information of this leaf node will send it to the prediction algorithm. The prediction algorithm will use the illness (situation) label to search through the cache memory for similar records. If there is a cache prediction that can match the label, the system will then use the records from this cache as prediction result. If there is no predictions found in the cache that can match the current label, the prediction algorithm will use the pointer value to search across the mapping table. It uses corresponding physical addresses to get these similar records for analysis. Here is an example of the mapping table. (see Table 3).

**Figure 4:**
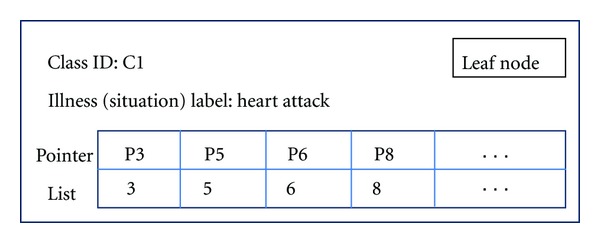
Pointer list in leaf node.

Every record has a unique pointer value which is created by the system in correspondence to its record ID. Suppose record ID (RID) is R1, then R1's pointer is P1 and its value is 1. When a new record is inserted into a database, its stored position (physical path) and the pointer value is recorded in the mapping table. The training function will use this new record to train the VFDT algorithm as the feedback process. Here we have an example of structure of pointer list in a leaf node as depicted in Figure 4.

### 2.2. VFDT Training Process and Searching Process

**Figure 5:**
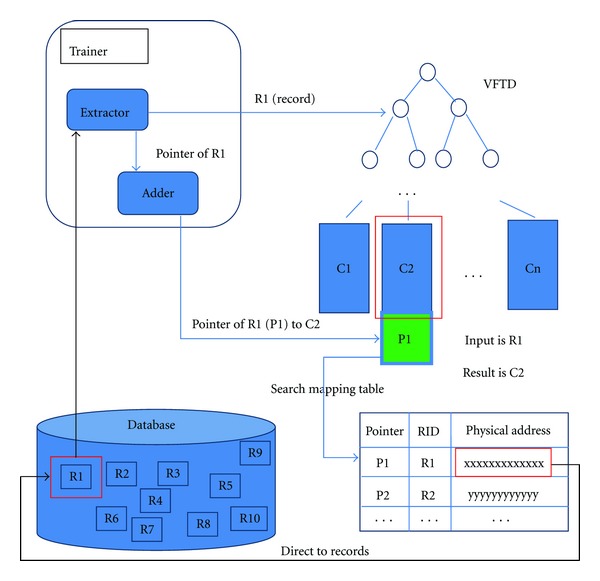
Training and searching process.

How the pointer is added to the VFDT's leaf nodes is described as follows. The pointer is added on the leaf node during the training process. When the training algorithm uses a series of medical records to train the VFDT, it will add the record's pointer to the result leaf node. The training process is run whenever there is a new medical record to be added into the database. For example the extractor in the training function wants to fetch a medical record R1 as a training data, then extractor sends R1's medical data to VFDT algorithm and R1's pointer to the Adder which is an internal data management function. When VFDT finishes the analysis of R1, it gives the result leaf node C2, the Adder adds a pointer P1 to the leaf node C2. When VFDT directs the current situation to a certain leaf node, the system will use the pointer stored in this leaf node to search across the mapping table. From the table, the physical address stored can be retrieved and subsequently it proceeds to collect the history record.  Figure 5 shows this concept—about how a pointer adds to a leaf node and searches for a certain record.

**Figure 6:**
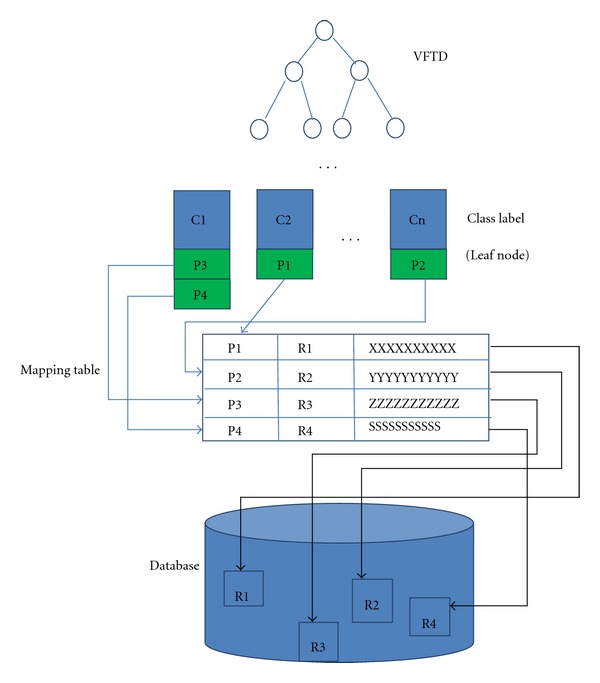
How mapping works in the initial training stage for VFDT.

At the initial stage, all medical records were stored in traditional way like shelves of archive. We use this library of existing medical records as initial data to train up the VFDT decision tree. It is well popularly known that VFDT decision tree needs many records to train up to a satisfactory classification performance. Once this incubation period is over, the decision tree model is adequately trained; classification can be used on the fly. The sensitivity of the VFDT that is related to how often it should readjust the rules under the tree so to reflect the updated relations of the new incoming to the actual rules can be configured by the user at will. During this initial training process, all medical records were added to a pointer. After initial training, one leaf node can represent one illness class and each leaf node can visit the medical records in their class directly.  Figure 6 show the VFDT, mapping and database after initial training process.

### 2.3. Prediction Process

**Figure 7:**
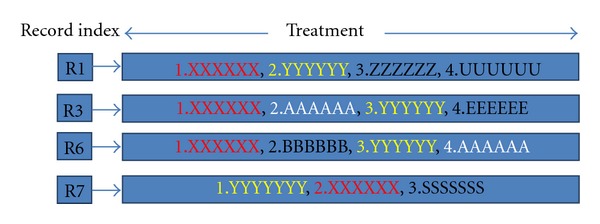
Extract most frequently used treatment from similar history records.

How the prediction algorithm works is introduced. When a classified result (leaf node) is sent to prediction algorithm, prediction will use pointers of this result (leaf node) to locate similar history of medical records. These records have similar medical data. So the common illness description of these records can be used for current situation. The description covers treatment, diagnosis, and illness history and they are written by different doctors. So these descriptions will have little difference in expression. For example, A : (heart attack, taking compound reserpine tablets) and B : (treatment is used to record history of treatment). So before we use frequency algorithm to find the most common description, we need an algorithm to find the description which has the same meaning and different expression. Fortunately, there are already many useful algorithms and applications in Natural Language Processing, and, in my system, we used the sentence similarity [8] and semantic similarity [9] to find the similar description for a certain kind of illness, then we marked the similar description with the same color and extract the most frequent description. History of treatment is used for medical advice against current illness. History of diagnosis will be used as the system diagnosis for current situation and history of illness description will be used for the prediction of the current situation. There is an example that shows how to extract the treatment information: suppose the leaf node points to R1, R3, R6, and R7. Then prediction will extract most frequency treatment actions. The linking of the leaf nodes and the treatment actions are shown in Figure 7.

**Figure 8:**
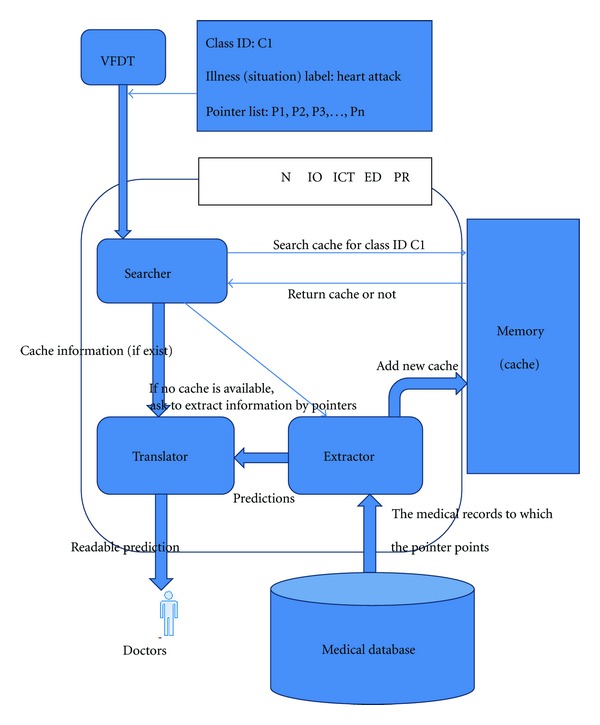
Prediction process.

For this example, the treatment made by the system will be in this shape: 1.XXXXXX 2.YYYYYY 3AAAAAA. Finally, prediction will use these extracted information as the result. And prediction will store this information in its memory as a cache. When the same classified result (leaf node) is sent for prediction, the prediction function will use the cache information directly. It can make the system more efficient. In order to keep up the accuracy of the system, system will clear the cache result when there are new pointers added to the match leaf node. The system will use the new pointer list to get the corresponding history record again, and then it extracts new result and stores it in memory as a new cache for this classified result (leaf node). For the process to extract useful information in database, it will not cost much time and system resource, because it is just a simple statistical method and it just considers the data in a relatively small data set (just consider the data which the current leaf node points to). So the extract process can run in real-time and will not delay the real-time decision.  Figure 8 show the process of the prediction.

### 2.4. Feedback Process

**Figure 9:**
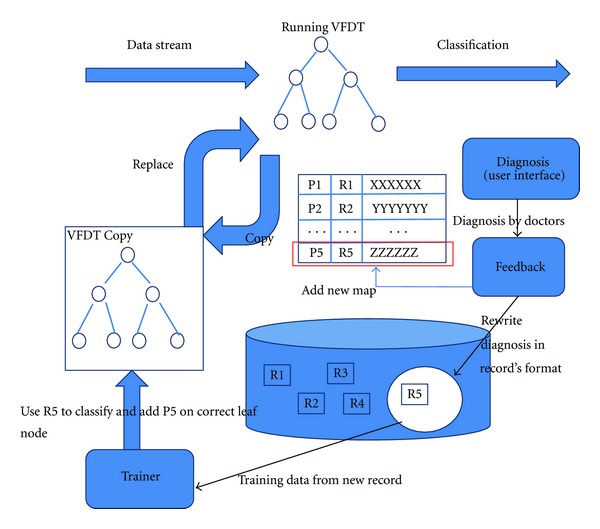
Feedback process.

The Feedback process is mainly used to update the mapping table and VFDT. When the system received a new diagnosis made by doctors, feedback function will rewrite the diagnosis into a uniform format and store it in a medical database. Feedback function will also add the pointer and physical address of this new record into the mapping table. Then the trainer will use this new medical record to train the VFDT tree. Trainer will first copy the running decision tree and train the copied one. In this way the training process will not affect the system running. When the train is finished, the pointer of this new record will be added to a correct leaf node and the system will do a frequency analysis for the new data set which this leaf node represented to get the latest treatment, diagnosis, and prediction information. Trainer will also replace the running decision tree by this new trained one. The operational flow of the feedback process is shown in Figure 9.

## 3. Comparison with IBM's

IBM lately published a paper in 2011 called “A system for mining temporal physiological data streams for advanced prognostic decision Support” [10]. In their paper, they designed a system that can monitor data streams from ICU and make a prediction. This system is perfect; it almost takes into all aspects. However, it needs an offline system to analyze the medical database using cluster and Locally Supervised Metric Learning (LSML). LSML is an algorithm to analyze the data class to find the representative data matrix for this class. LSML calculates the distance between patients' history records (medical data stored in matrix) and the formula is

(1) dm(xi,xj)  =  (xi−xj)TP(xi−xj).

We know that to compute large amounts of distance between two points will cost a lot of system resources. LSML needs to compute large amounts of distances between matrixes (multidimensionalpoint), so it needs a lot of system resources and time. It is therefore speculated that they designed an offline system running independently from the online system. In our design, there is no need for offline systems; all the systems are online and real-time. In our design, the cluster process and initial training process will be finished at same time. The most complex calculations are just to update the VFDT (decision tree). So the proposed system requires much less system resources than IBM's. However, the disadvantage in our design is that the initial system needs a process of initial training. Although, it may not be a big problem, it spends some time on training before it is acceptable. IBM's system also needs clustering process and this process is also to operate offline. After training, the VFDT can classify the data stream and direct the current situation to similar medical record sets. The new diagnosis made by doctor will be a new medical record and it can be fedback to system. VFDT will use this new record to update (training) itself and once VFDT finished the update, the data class in database will be updated naturally. As the VFDT can update its decision tree very quickly, the update process can run online (real-time).  Table 4 compares our design and IBM's.

## 4. Conclusion

In this paper, a new system is introduced that can analyze medical data streams and can make real-time prediction. This system is based on stream mining algorithm called VFDT. The VFDT is extended with the capability using pointers to allow the decision tree to remember the mapping relationship between leaf nodes and the history records. This way can save the need of the offline clustering process that reduces the resource consumption for the system. In fact, the clustering process and initial training in theory can be processed together at the beginning. After initial training, from any leaf node we can use its pointer list and mapping table to retrieve the corresponding history records directly. It is suggested that clustering and classification should be used together for a more accurate prediction—prediction of a new insight requires retrieving the past cases that are represented as similar history records. Clustering helps grouping them, similar ones together. Prediction helps locating them subsequently. The design as advocated in this paper is at its initial phase and all these design elements are in conceptual stage. A system prototype is currently being developed so that soon experiments with suitable medical data can be performed in order to validate the efficacy of the design model. The merit of the proposed system is that it runs online in real-time which can be portable and operate in mobile scenario on the move. It will be useful especially for emergency help or in field rescue missions.

## Figures and Tables

**Table 1:** Existing clinical decision support systems.

Name	Author/source	Based on
A decision tree for tuberculosis contact investigation [3]	Gerald LB, Tang S, Bruce F et al., Am J Respir Crit Care Med 2002; 166: 1122–1127	Traditional decision tree
Iliad [4]	Developed by University of Utah School of Medicine's Department of Medical Informatics	Bayesian network
An artificial neural network ensemble to predict disposition and length of stay in children presenting with bronchiolitis [5]	Walsh P, Cunningham P, Rothenberg SJ, O'Doherty S, Hoey H, Healy R.	Neural network
MYCIN [6]	Developed at Stanford University by Dr. Edward Shortliffe in the 1970s	Rules
BioStream: a system architecture for real-time processing of physiological signals (data stream mining, focus on detection) [7]	Amir Bar-Or, David Goddeau, Jennifer Healey, Leonidas Kontothanasis, Beth Logan, Alex Nelson, JM Van Thong	Physical data stream detection QRS (the algorithm was not described clearly in original paper)

**Table 2:** Defects of traditional implementations.

Algorithm	Defect
Traditional decision tree	Only can analyze static and finite data set. Cannot handle data stream
Bayesian network	Difficulty to get the probability knowledge for possible diagnosis and not being practical for large complex systems given multiple symptoms
Neural network	Training process consume so much time that users cannot use the systems effectively
Rules	It is difficult for experts to transfer their knowledge into distinct rules, and it needs many rules to make system effectively

**Table 3:** Mapping table example.

Pointer	RID	Physical address
P1	R1	00000C900000FFFF
P2	R2	000B80000000FFFF
…	…	…

**Table 4:** Comparison between IBM's system and our design.

	IBM	My design
Need offline analysis	Yes	No
System resources	Offline analysis (LSML) needs to compute the distance between matrixes and this process will cost a lot of resources	No need to do complex calculations, the most complex calculation is just the update of VFDT
Need training	No, but it also needs analysis of the database before formal using (for cluster the records in database)	Yes, before formal use it needs initial training
